# Editorial: NRF2 signaling pathway: New insights in the field of reno-cardiovascular health

**DOI:** 10.3389/fphar.2023.1193005

**Published:** 2023-04-17

**Authors:** Zeba Farooqui, Juan Antonio Moreno, Alfonso Rubio-Navarro

**Affiliations:** ^1^ College of Pharmacy, Heart and Kidney Institute, University of Houston, Houston, TX, United States; ^2^ Maimonides Biomedical Research Institute of Cordoba (IMIBIC), Hospital Universitario Reina Sofía, Córdoba, Spain; ^3^ Department of Cell Biology, Physiology and Immunology, University of Cordoba, Cordoba, Spain; ^4^ Biomedical Research Networking Center on Cardiovascular Diseases (CIBERCV), Madrid, Spain; ^5^ Instituto de Investigación Biosanitaria de Granada (ibs.GRANADA), Granada, Spain; ^6^ Laboratory of Advanced Therapies (CTS-963): Differentiation, Regeneration and Cancer, Center of Biomedical Research, University of Granada, Granada, Spain; ^7^ Excellence Research Unit “Modeling Nature” (MNat), University of Granada, Granada, Spain

**Keywords:** NRF2, oxidative stress, inflammation, renal disease, cardiovascular disease

An excessive generation of reactive oxygen species (ROS) induces oxidative stress and subsequent inflammation, and is considered major drivers in the pathogenesis of renal and cardiovascular diseases (CVDs) (Dounousi et al., 2006; Dubois-Deruy et al., 2020). Nuclear factor erythroid 2-related factor 2 (NRF2) is a transcription factor that plays a major role in regulating the expression of a wide range of antioxidant and cytoprotective genes and is regarded as a vital pharmacological target in treating renal and cardiovascular abnormalities (Ma, 2013). Therefore, there is a growing interest in understanding the role of NRF2 signaling in oxidative stress-related disease progression and identifying novel approaches targeting NRF2 that may lead to the implementation of clinical treatments that improve the care of patients with renal diseases and CVDs.

This Research Topic contains 6 papers, including 5 original research articles and 1 review, reporting relevant information about the key role of NRF2 in the context of renal diseases and CVDs.

Oxidative stress and inflammation have been identified as crucial in most of the key steps in the pathogenesis of CVDs (Dubois-Deruy et al., 2020). In this sense, NRF2 has been postulated as a promising therapeutic target against CVDs through the induction of the expression of several antioxidant and anti-inflammatory genes (Li et al., 2009). The original article presented by Gonzalez-Carnicero et al., have reported that NRF2 activation prevents the pro-oxidant and pro-inflammatory effects induced by Interleukin-1β in vascular smooth muscle cells through downregulation of Toll-like receptor 4 (TLR4)-dependent pathway.

Diabetes *Mellitus* (DM) is characterized by an increased risk of developing CVDs, mostly attributed to the adverse effects of hyperglycemia and oxidative stress on vascular and endothelial cells (Avogaro et al., 2011). In the article entitled “Effect of sulfasalazine on endothelium-dependent vascular response by the activation of NRF2 signaling pathway” by Sonmez et al., the authors investigated the effects of sulfasalazine on endothelial dysfunction in the context of DM*.* Treatment with sulfasalazine reduced oxidative stress and cell injury induced by high glucose concentrations using isolated organ bath experiments. Molecular analyses revealed that the therapeutic potential of sulfasalazine was a consequence of inducing NRF2/HO-1 activity via the ERK and JNK pathways.

Several studies have reported that a variety of natural products show protective effects against oxidative stress by activating NRF2 signaling pathway (Tavakoli et al., 2021). In this sense, activation of NRF2 using natural products provides an opportunity for the treatment of renal diseases and CVDs. In this Research Topic a study developed by Wu et al., reported that tanshinone, a natural compound extracted from *Salvia miltiorrhiza* protected against oxidative stress-induced myocardial injury in mice. *In vitro* and *in vivo* models revealed tanshinone I reduced cardiomyocyte oxidative stress and subsequent apoptosis through NRF2 activation and MAPK signaling inhibition, indicating the potential therapeutic application of tanshinone I for oxidative stress–induced CVDs.

The protective effect of natural products against cardiac injury has also been evaluated by Li et al., in this Research Topic. Doxorubicin, an antibiotic widely used in the treatment of cancer, induces cardiotoxicity, limiting its clinical applications (Ewer and Ewer, 2015). This original research shows that Fisetin, a natural flavonoid that is abundantly present in fruits and vegetables, promotes cardioprotective effects against *in vivo* and *in vitro* doxorubicin-induced cardiotoxicity by reduction of ferroptosis. Authors revealed that Fisetin reduces malondialdehyde levels, a biomarker of oxidative stress induced lipid peroxidation and increases glutathione levels through SIRT1/NRF2 signaling pathway activation. Overall, these studies indicate that natural products such as diterpenoids and flavonoids can be effective therapeutic tools against cardiotoxicity induced by oxidative stress.

Gentamicin is an aminoglycoside antibiotic used to treat potentially severe Gram-negative bacterial infections. However, the therapeutic use of gentamicin is frequently restricted by the induction of nephrotoxicity (Quiros et al., 2011). Gentamicin-induced kidney injury is associated with increased oxidative stress and inflammation that ultimately lead to tubular cell death and inflammatory cell infiltration. In this Research Topic, Althunibat et al., investigated the renoprotective effects of formononetin, a natural isoflavone found in several plants with anti-oxidant, anti-inflammatory, anti-apoptotic, and tissue-protective properties. They showed that formononetin prevent kidney injury and improves renal function in rats treated with gentamicin by decreasing lipid peroxidation and enhancing antioxidant capacity via NRF2/HO-1 axis.

Finally, the article submitted by Wu et al., reviewed the potential of NRF2 as a therapeutic target for the management of CVDs, focusing on its role in hypertension, atherosclerosis, myocardial ischemia, heart failure, peripheral artery disease, rheumatic heart disease, and other cardiomyopathies. In addition, the authors summarized the potential of different natural products and their molecular mechanisms targeting the NRF2 signaling pathway in protecting against CVDs.

In summary, the Research Topic “*NRF2 Signaling Pathway: new Insights in the Field of Reno-Cardiovascular Health*” has collected different studies focusing on the role of NRF2 in the pathophysiology of kidney and cardiovascular system and summarizing the impact of NRF2 signaling pathway as a therapeutic target. We hope that this Research Topic will improve our understanding of the molecular mechanisms of NRF2 and provide broad insights for future therapies in the context of reno-cardiovascular diseases.

## Limitations

Since our topic has focused on the importance of NRF2 signaling pathway in reno-cardiovascular health, the topic’s scope is restricted to targeting NRF2 transcription factor in renal diseases and CVDs. In the editorial, we have summarized the 6 articles that are published under the Research Topic. We anticipate the published articles in this Research Topic to be of interest to the readers and expect researchers to benefit from achieving further progress in understanding the role of NRF2 signaling mechanisms underlying renal diseases and CVDs and the development of novel therapeutics. However, considering the importance of NRF2 in the regulation of expression of several antioxidants and cytoprotective proteins and enzymes, it is worth investigating the role of this transcription factor in other physiological and pathological conditions.
